# Two Coordinates for Dopamine in the Basal Ganglia

**DOI:** 10.3390/neurosci7050104

**Published:** 2026-09-24

**Authors:** Szatmár Horváth, Károly Mirnics

**Affiliations:** 1 Toronto Private Hospital, Aurora Health, Toronto, NSW 2283, Australia; shorvath@volunteer.unmc.edu; 2 Munroe-Meyer Institute, University of Nebraska Medical Center, Omaha, NE 68106, USA

**Keywords:** striosome and matrix, dopamine receptors, medium spiny neurons, corticostriatal projection neurons, basal ganglia, action selection, Parkinson’s disease, schizophrenia

## Abstract

The striatum is the principal input structure of the basal ganglia, central to selecting behaviour and learning from its consequences. Its output is conventionally described through D1-associated direct and D2-associated indirect pathways. Striosome–matrix organisation provides a second, intersecting division. Together, these two divisions define four principal striatal output populations: matrix D1, matrix D2, striosomal D1 and striosomal D2. Mouse studies identify striosomal pathways that regulate dopamine neurons alongside the canonical pathways controlling basal ganglia output. We propose that these populations perform complementary functions: matrix routes predominantly support behavioural selection, while striosomal routes shape the dopamine signal that influences selection and learning. The key principle is that a receptor’s functional significance depends on its circuit position. Inhibiting a basal ganglia output neuron and inhibiting a dopamine neuron have different consequences, even when the cells defining each route express the same dopamine receptor. This distinction connects pathway anatomy with parallel basal ganglia loops, dopamine coding and a proposed relative weighting of content and value across compartments. The two coordinates describe preferential circuit organisation within a broader diversity of cell types, overlapping connections and regional specialisations. Parkinson’s disease and schizophrenia illustrate how neuronal degeneration and circuit dysregulation affect different parts of this organisation, while systemic treatments act beyond the circuits requiring correction. The framework yields specific predictions about pathway connectivity, compartmental information processing and dopamine feedback, providing an experimentally testable link between cellular organisation, behaviour and clinical function.

## 1. Introduction

Pharmacological treatment of movement disorders and psychosis reveals an intriguing symmetry in dopamine function. Dopaminergic treatment can improve movement in Parkinson’s disease while promoting impulse control problems or psychotic symptoms in susceptible individuals. Conversely, antipsychotic D2 receptor blockade can relieve psychosis while producing bradykinesia and rigidity [1,2,3]. These effects do not make Parkinson’s disease and schizophrenia opposite versions of one disorder. They show that a therapeutic change in dopamine signalling can benefit one circuit while disturbing another.

The familiar direct–indirect pathway model explains an essential part of this organisation. Most striatal spiny projection neurons (SPNs) express predominantly D1 or D2 dopamine receptors. D1 receptors couple principally to Gs/Golf proteins and promote cyclic AMP signalling; D2 receptors couple to Gi/o proteins and reduce it. D1-enriched direct pathways inhibit the internal globus pallidus (Gpi) and substantia nigra pars reticulata (SNr), thereby disinhibiting downstream targets. D2-enriched indirect pathways act through the external globus pallidus (Gpe) and associated circuitry to regulate those same output nuclei [4,5,6,7]. Receptor identity thus identifies a major dimension of striatal organisation but does not exhaust it.

A second dimension distinguishes striosomes from the surrounding matrix. Defined initially by their neurochemistry, these compartments differ in afferent weighting, intrinsic physiology and output connections [8,9,10,11,12,13,14]. Both contain D1- and D2-expressing neurons. Recent work in mice resolves striosomal D1 and D2 pathways that exert opposing effects on nigral dopamine and movement [15]. The resulting question is functional: what changes when the direct–indirect distinction is read together with compartment identity?

We propose that compartment and receptor identity should be treated as intersecting coordinates. Matrix routes predominantly support the selection and implementation of behaviour, while striosomal routes regulate the dopamine signal that guides selection and learning. We describe these complementary roles as actor and controller, adapting terminology from computational models of reinforcement learning. The distinction rests on their downstream targets: inhibiting a basal ganglia output neuron releases its targets from inhibition, whereas inhibiting a dopamine neuron changes the signal returning to the striatum. This Perspective develops the functional consequences of that difference, linking how neurons integrate incoming information to regional circuit specialisation and the benefits and limitations of treatment.

This synthesis brings together five traditions: dopamine deficiency and receptor blockade, parallel loops, direct and indirect pathways, striosome–matrix organisation, and dopamine coding [16,17,18,19,20,21,22,23,24,25,26,27]. Table 1 identifies what each contributes. The four principal compartment–receptor combinations provide the anatomical starting point; their proposed computational roles and clinical applications generate separate, testable predictions. What is novel here is not the individual findings, most of which are established, but their integration: reading compartment together with receptor identity yields four routes rather than two and makes the behavioural meaning of a receptor depend on the route it enters rather than on receptor identity alone. This reframes the direct–indirect model, striosome–matrix organisation, parallel loops and dopamine coding as complementary projections of one architecture.

## 2. Two Coordinates Define Four Principal Populations

Compartment and receptor identity classify striatal neurons in two intersecting ways (Figure 1). The matrix contains both D1 and D2 SPNs, and so do striosomes, although their proportions vary across subtypes and territories [28,29,30]. We describe these coordinates as orthogonal because knowing a neuron’s compartment does not specify its receptor identity, and vice versa. This describes how the two classifications intersect, without implying equal population sizes, statistical independence or an exhaustive four-type taxonomy.

The compartments and the D1/D2 segregation that crosses them were first defined largely in rat and cat studies [9,12,13]; recent mouse work now resolves the four principal routes. Mouse experiments tracing specific projections give this organisation a functional anatomy. Striosomal D1 neurons preferentially innervate dopamine neurons in the substantia nigra pars compacta (SNc), including their dendrites descending into the SNr region. Striosomal D2 neurons preferentially engage a central GPe zone connected to dopamine neurons. The corresponding matrix populations predominantly engage basal ganglia output circuitry: D1 neurons project to the entopeduncular nucleus—the rodent GPi homologue—and SNr, while D2 neurons project to a more peripheral GPe territory [15]. These four principal routes have been characterised most fully in mice using Pnoc- and Nts-enriched populations as proxies for the striosomal D1- and D2-biased pathways [15].

The functional importance of this arrangement becomes clear when the pathways are activated. Stimulating striosomal D1 populations reduces striatal dopamine and locomotion, whereas stimulating striosomal D2 populations increases both [15]. These effects reverse the behavioural signs conventionally associated with the direct and indirect pathways. Other manipulations of striosomal circuitry also demonstrate its capacity to inhibit dopamine release and movement [31,32]. Together with the established matrix pathways, these findings show why receptor identity must be interpreted in its circuit context: cells expressing the same receptor can occupy different positions in the feedback system.

As shown in Figure 1a, thalamocortical projections reach different cortical layers and dendritic compartments [33,34]. Calbindin-rich thalamic populations contribute superficial projections, whereas parvalbumin-rich core relays preferentially engage middle layers; this segregation is relative and more graded in primates than in rodents. In the mouse prefrontal cortex, ventromedial input strongly engages PT apical dendrites, and local layer-1 circuitry influences its effect [33]. Basal and apical activity can interact at the calcium spike initiation zone, labelled the Ca^2+^ nexus [35]. The proposed value weighting of this interaction remains a functional hypothesis.

In Figure 1b, the coronal section shows striosomes within the surrounding matrix, with an inset detailing their preferential inputs and outputs [9,12]. The PT-to-striosome and IT-to-matrix labels represent preferential routing in the proposed synthesis; both cortical projection classes can innervate both compartments [30]. The inset also distinguishes CM/Pf input to dendritic shafts from IT input to distal spines. The striosome-to-SNc inhibitory connection summarises the direct route; Figure 2 distinguishes this D1 route from the relayed D2 route. The territorial gradient indicates regional variation without assuming identical compartmental organisation in every territory.

As shown in Figure 1c, striosomal axons form bouquets around descending dopamine neuron dendrites, while matrix pathways reach basal ganglia output nuclei [12,36]. Nigrostriatal projection preferences contribute to compartmental feedback [37]. Inhibitory SNr collaterals contact dopamine neurons [38,39], providing a route through which striatal inhibition of SNr could disinhibit dopamine [40]. SNr output to thalamic and tectal targets is also inhibitory. Coordinated striosomal and matrix-associated input onto the same identified dopamine neuron remains a prediction.

Striosomal and matrix pathways have distinct projection preferences, but their targets overlap. Single-neuron tracing in rats reveals both shared targets and specialised nigral projections, while individual primate axons can branch to several pallidal and nigral nuclei [29,41]. Dopamine neurons receive input from the matrix as well as patch and exo-patch populations, and direct matrix input can inhibit SNc neurons [30,42]. The matrix can therefore influence dopamine both directly and indirectly through SNr neurons. The functional distinction between compartments lies in the pattern and relative strength of these connections.

Molecular mapping reveals further diversity within this organisation. The principal SPN classes contain spatially distinct populations [43], and human striatal subregions contain D1 and D2 populations in both striosomes and the matrix, alongside outlier populations and regional gradients in gene expression [44]. Compartment and receptor identity thus provide two organising coordinates within a broader cellular landscape. Regional molecular differences refine this organisation and must be considered when comparing circuits across species.

A separate population of SPNs coexpresses D1 and D2 receptors, projects to GPe and can modulate the motor effects of the principal D1 and D2 populations [45]. These cells add another component to the circuit and require separate analysis. They should also be distinguished from the receptor-biased outlier populations identified by molecular mapping, which are not defined by D1/D2 coexpression. The four principal combinations therefore organise a major part of the system, while additional populations can connect or modify their operation.

## 3. The Route Sets the Sign

Receptor identity describes how a neuron responds to dopamine. Its route describes what that neuron acts on. Both are needed to understand the consequences of its activity within the circuit (Figure 2).

In the canonical matrix D1 route, inhibition of GPi/SNr releases downstream thalamic and brainstem targets from inhibition. In the canonical matrix D2 route, inhibition of GPe recruits circuitry that can increase inhibition from basal ganglia output nuclei. These operations contribute to selecting and constraining behaviour [4,5,6,7,40]. They function as interacting population processes: direct and indirect pathway populations are both active around action initiation [46].

Striosomal routes instead regulate dopamine neurons. Inhibition from striosomal D1 neurons suppresses dopamine output while it is active. Striosomal D2 neurons inhibit an inhibitory pallidal relay, which can disinhibit dopamine neurons [15]. The same receptor label can therefore have different behavioural implications across compartments. Whether a route ultimately suppresses or facilitates activity depends on its connections and the timing of its activation, while its behavioural effects depend on the wider network.

Compartment identity also influences how SPNs themselves respond to dopamine. D1- and D2-linked signalling regulate excitability and synaptic plasticity through different intracellular mechanisms [47,48]. The D1–cAMP–PKA cascade provides the substrate on which dopamine writes plasticity, whereas D2 signalling attenuates it, both intracellularly and by suppressing prefrontal input onto the same neurons [47,48,49]. Even within D1-expressing populations, the response varies by compartment: in mouse recordings, D1 receptor activation prolongs evoked up-states in matrix direct pathway neurons but shortens them in striosomal direct pathway neurons [50]. Direct stimulation of a D1-expressing population therefore need not reproduce the effects of activating its D1 receptors. Together, these findings show that the effects of receptor signalling depend on the physiology of the neuron, just as the consequences of its activity depend on its downstream targets.

Matrix and striosomal pathways may also converge on dopamine neurons through nigral feedback. Striosomal terminals form dendron bouquets around descending dopamine neuron dendrites [36,51]. SNr projection neurons have inhibitory collaterals that contact dopamine neurons, allowing striatal inhibition of SNr to disinhibit dopamine neurons [38,39]. Circuit-specific experiments demonstrate regional striatonigrostriatal feedback, including strong closed-loop organisation [52], extending earlier physiological observations [53,54].

We propose that direct striosomal inhibition and matrix-associated disinhibition through SNr jointly regulate selected dopamine neurons. The component pathways have experimental support; their coordinated convergence from identified compartments onto the same neuron is a specific prediction of the framework. Direct matrix inputs, branching projections and additional pallidal routes also contribute, with the net response depending on the relative strength and timing of these inputs. Within this interconnected network, influencing basal ganglia output and regulating dopamine remain distinct, complementary functions.

## 4. A Pause Can Become a Teaching Transient

Controlling dopamine involves more than setting its average level. Learning depends on when a signal arrives, how it changes and which synapses remain eligible for modification. Striosomal inhibition provides a mechanism for shaping this timing.

Comparisons of genetically identified striosomal, matrix and pallidal inputs onto mouse SNc neurons reveal distinct patterns of inhibition. Striosomal input produced particularly strong inhibition that became stronger during repeated stimulation and recruited a prominent GABA_B_ component. Release from this inhibition could generate rebound firing. Pallidal inputs preferentially engaged GABA_A_ receptors at somatic and proximal dendritic sites and did not produce the same rebound under the tested conditions [42]. Figure 3 illustrates this physiological distinction and its proposed role in learning.

Synaptic location helps explain the difference. Striosomal axons contact descending nigral dendrites, where GABA_B_ signalling can generate sustained potassium conductance [36,42,55,56]. GABA_A_ and GABA_B_ inhibition differ in their reversal potentials and kinetics, and dopamine neurons have distinctive chloride regulation [57,58]. Sufficient hyperpolarisation can recruit intrinsic rebound mechanisms, including T-type calcium channels in susceptible ventral tier neurons [59]. The importance of GABA_B_ signalling is supported by targeted knockdown of Gabbr1 in ALDH1A1-positive nigral dopamine neurons, which reduces inhibition and abolishes the rebound and locomotor effects evoked by Kremen1-positive SPNs [60].

A pause followed by rebound therefore offers a plausible way to transform feedback into a temporally structured dopamine signal. Bursts can increase dopamine release disproportionately [61,62], while experiments on D1-expressing SPNs show that dopamine promotes structural plasticity within a brief window following glutamatergic activity [63]. Dopamine-dependent plasticity also differs between D1 and D2 populations [48]. We therefore predict that the phase and timing of striosomal feedback influence which active corticostriatal synapses are modified. The direction of plasticity should be measured separately in D1 and D2 populations.

Other circuits also shape the timing and pattern of dopamine release. Disinhibition can support bursting through circuit mechanisms [64], and striatal cholinergic interneurons can trigger dopamine release locally at terminals [65]. Striosomes therefore contribute to dopamine control within a distributed system. Their circuit position allows selected striatal populations to shape the dopamine signal that guides subsequent behaviour and learning.

## 5. What Each Compartment Reads

The different outputs of matrix and striosomal pathways suggest that they may also place different weights on incoming information. A candidate action or representation has content—its identity, timing and available response—and value—its expected benefit, cost, urgency or contextual significance. These properties can vary independently. We propose that matrix populations place greater weight on content, while striosomal populations place greater weight on value.

This proposal reflects differences in the relative strength of inputs to each compartment. Corticostriatal tracing in rodents and cats reveals distinct compartmental input patterns, while macaque orbitofrontal and prelimbic regions preferentially innervate striosomes [13,66,67,68]. Thalamostriatal projections also differ: the centromedian–parafascicular complex preferentially innervates the matrix in squirrel monkeys, and midline thalamic inputs contribute a complementary compartmental organisation in cats [69,70]. These findings support differential weighting within a system of overlapping sensory and contextual inputs.

Behavioural studies provide a functional basis for this distinction. Compartment-resolved recordings reveal combined representations of actions and outcomes during cost–benefit foraging [71]. Manipulating a corticostriatal pathway that preferentially targets striosomes changes decisions under conflict, and striosomal manipulations influence value-based learning [72,73]. In macaques, stimulation of prefrontal sites associated with striosomal projections biases approach–avoidance decisions [74]. Together, these results support different contributions to evaluating and implementing choices.

Differences in how cortical neurons integrate information offer one possible mechanism for this weighting. Intratelencephalic (IT) neurons connect telencephalic structures; pyramidal tract (PT) neurons also project to subcerebral targets and send corticostriatal collaterals [75,76,77]. Deep-layer pyramidal neurons can combine basal and apical inputs nonlinearly, allowing distal input to modify the effect of input near the soma [35,78]. In the mouse prefrontal cortex, thalamic inputs engage distinct layer-1 circuits and influence the apical dendrites of different projection-defined populations [33]. Dendritic geometry and integration further shape how incoming activity affects neuronal output [79,80].

We propose that some PT circuits emphasise how context shapes a candidate’s influence on output, while IT circuits retain more detail about the available candidates. Tracing nevertheless reveals substantial overlap in the cortical origins of IT-type and PT-type inputs to the two compartments [30]. Projection class, cortical layer and compartment therefore describe related population biases whose strength must be measured in the relevant region and task. Shared inputs can acquire different effective weights through dendritic integration, synaptic strength, local inhibition and learning. These proposed IT/PT weightings are working hypotheses that may vary across regions, species and tasks. The thalamic calbindin–parvalbumin distinction is likewise relative and more graded in primates than in rodents [34].

We use the term registration for this appropriate association of content with value: the assignment of significance to the representation that warrants it. Integration of apical and somatic inputs offers one possible cellular mechanism for this association. Box 1 illustrates the concept without assuming a one-to-one correspondence between an experience and a neuronal class.

Box 1 **One ring, one architecture.**

Your partner declined the invitation to the opera, so you have come alone, the seat beside you empty. You are absorbed in Verdi’s *Otello* when your telephone rings.

Two things are **registered** separately. **Content** identifies what happened: your telephone is ringing, from your coat, specifying a reach and a press. Value marks what it is worth—here, it is the context that sets it: the interrupted performance, the silence, the audience around you. Neither is sufficient alone. The ring is not embarrassing, and simply being in the theatre gives you nothing to be embarrassed about. Put them together—your telephone ringing here, now, during the performance—and deep embarrassment follows. At home, the same ring might barely matter. Here, context gives it **value**, changing the **situated representation** from *my telephone is ringing* to *I am interrupting the performance*.

That conjunction is handled in two ways at once, doing almost opposite jobs. One compresses information into a decision. The ring affords *I could silence it*; embarrassment makes that possibility worth acting on. When **content** and value coincide strongly enough, a second threshold is crossed: *I could silence it* becomes *silence it now*. Detail is traded for decisiveness.

The parallel process does the opposite: it preserves detail rather than collapsing it into a decision. The telephone, performance, turning heads and available reach remain active and distinguishable. The two must run together: one commits to action while the other preserves exactly what is being acted on.

Yet *silence it now* does not move your hand. You are already watching *Otello*; you could freeze, ignore the ring or leave. These are competing behavioural programmes. One must be released while the others remain blocked. The balance shifts in opposite directions: the reach gains control as its competitors lose it. Only then does intention become behaviour—attention leaves the stage; your hand enters the coat and the telephone falls silent.

Dopamine helps set that balance, while **value** shapes dopamine itself. Within milliseconds, the signal can fall and rebound above its usual level. You experience no two-step event; you simply interrupt one behaviour and execute another. But the brief swing arrives while the detailed representation is still active, allowing opposing pathways to update it differently. This may help explain why emotionally intense moments leave such vivid memories: while one process compresses the moment into *act now*, the other preserves its details as the teaching signal arrives. Later, a single detail—the opening bars of *Otello*, the darkened auditorium, another telephone ring—may bring the episode back with surprising force, so that memory, emotion, bodily response and readiness to act return together.

The same logic can arbitrate movements, words, faces, plans or thoughts. In Parkinson’s disease, *silence it now* may be fully formed, yet the reach fails to gain release. In psychosis, action may remain intact while context binds **value** to the wrong **content**. Your partner is absent; *Otello* surrounds you with jealousy and suspected infidelity; a telephone rings across the auditorium. An ordinary sound becomes *perhaps my partner is unfaithful; perhaps this is about me*. The sound has not changed. Its **value** has—and with it the **situated representation**. A neutral event has become evidence before reasoning can ask whether it was evidence at all.

## 6. A Common Organisation, with Regional Specialisation

Actor–critic models have long linked selection to evaluation and proposed that matrix and striosomes support these complementary functions [81,82]. The present framework extends that tradition by crossing compartment with receptor identity and specifying how the resulting routes influence different targets. The controller is deliberately broader than the critic: striosomal feedback can regulate dopamine dynamics without necessarily computing a complete reward prediction error on its own. Reward, movement, cost and context can contribute to this distributed computation [25,71,72,73]. Within this framework, dopamine links the selection of current behaviour to the modification of synaptic weights that shape future choices.

Parallel basal ganglia loops organise these operations across functional territories [23,83]. In sensorimotor circuitry, a candidate may be an overt action; in associative circuitry, it may be a rule, plan or internally generated representation [84,85]. Selection and evaluation are relevant to each domain, so matrix function extends beyond movement and striosomal function beyond limbic processing.

A common organising principle can take different forms across striatal regions. Compartment boundaries, receptor proportions, input distributions and molecular identities vary across the striatum [28,43,44]. Mouse mapping also identifies a dorsolateral striosome-free territory and a different caudal compartmental organisation [28]. Applying the framework to the ventral striatum likewise requires attention to its distinct molecular organisation and connections. Regional organisation determines which information these circuits process, while local molecular properties and connections shape how they operate. Regional variation is therefore an integral part of the framework.

## 7. Clinical Implications and Hypotheses in Parkinson’s Disease and Schizophrenia

We use the two disorders as contrasting illustrations of the architecture rather than as complete mechanistic accounts. The clinical formulations below are hypotheses the framework generates and organises; they are consistent with existing data but are not established disease mechanisms, and each is stated as a proposal to be tested.

### 7.1. Parkinson’s Disease: Loss of Dopamine and of the Circuitry Supplying It

Parkinson’s disease is a progressive neurodegenerative disorder. Its dopamine-responsive motor features reflect substantial loss of nigrostriatal dopamine innervation, with particularly severe involvement of ventrolateral nigral neurons and their sensorimotor striatal projections [86,87,88,89]. Dopamine depletion reduces D1-linked support of direct pathway activity and removes D2-mediated modulation of indirect pathway neurons. The resulting changes in excitability, plasticity and coordinated population activity impair movement selection and scaling [4,5,90,91].

The two-coordinate framework highlights a second consequence of neuronal loss: disruption of the pathways through which striatal feedback regulates dopamine. ALDH1A1-positive ventral nigral neurons provide a link between this feedback mechanism and neuronal vulnerability. Human tissue demonstrates vulnerability among these neurons, with related evidence from a macaque model [92,93]. In mice, ventral dopamine neurons receiving strong striosomal inhibition show pronounced rebound properties [42]. Ablation of ALDH1A1-positive nigrostriatal neurons impairs both locomotion and motor skill learning. Levodopa improves walking speed but does not restore motor skill learning; systemic administration of a D1 receptor agonist also fails to rescue the learning deficit [94].

These findings distinguish replacing dopamine or stimulating its receptors from restoring the cells that regulate its delivery. Within the framework, neuronal loss can disrupt behavioural selection and dopamine feedback together, motivating direct tests of compartmental feedback in Parkinson’s disease. Cognitive decline, axial impairment and nonmotor features also involve degeneration beyond the nigrostriatal system, including non-dopaminergic circuits [95].

### 7.2. Schizophrenia: Representation and Reinforcement Can Become Misaligned

Schizophrenia involves distributed circuit abnormalities and heterogeneous dopamine dysfunction. Human imaging most consistently identifies elevated presynaptic dopamine function in associative striatal territories, rather than a uniform increase throughout the striatum [96,97]. D2-sensitive signalling is an important therapeutic target, but the effects of receptor blockade do not establish a primary lesion of D2 SPNs, and dopamine elevation is not universal across clinical subgroups [98].

Cortical and thalamocortical abnormalities may also alter the information entering striatal circuits. Post-mortem studies report altered presynaptic gene expression and reduced dendritic spine density in prefrontal pyramidal neurons, while other work identifies abnormalities of cortical inhibition [99,100,101]. Imaging demonstrates altered thalamocortical connectivity, including reduced prefrontal and increased sensorimotor coupling [102,103].

These findings constrain the clinical hypotheses without establishing their cellular implementation. The PET findings cited here concern regional striatal dopamine function and do not identify dysfunction within individual striosomes or the matrix, nor do they establish a causal PT–striosome mechanism. The cited spine density study concerns basilar dendrites of prefrontal layer-3 pyramidal neurons [100]; it does not demonstrate a lesion of layer-5 PT apical dendrites. These observations therefore support investigation of distributed circuit dysfunction while leaving the proposed registration mechanism to be tested directly.

Within the proposed framework, unreliable representations and inappropriate reinforcement are separable but interacting problems. A representation can be distorted before it reaches the striatum; an otherwise ordinary representation can also acquire disproportionate significance through dysregulated dopamine signalling. Their interaction provides a circuit formulation of aberrant salience [26,27]. This extends the tonic–phasic account [24] by considering what is represented when dopamine acts, alongside the amount and timing of its release. Dopamine dysfunction need not be the initiating disturbance.

Here, failed registration occurs when significance is assigned inappropriately to a representation. We predict that experimentally mismatching a representation and its contextual value can alter subsequent choice even without a general increase in dopamine. Disrupted apical integration could contribute to this mismatch, while recurrent cortical, thalamic and striatal mechanisms could produce the same computational error. Parkinson’s disease and schizophrenia thus illustrate how neuronal loss and dysregulated information processing can disturb behavioural selection and dopamine feedback in different ways.

## 8. Why Treatment Benefits One Circuit and Burdens Another

Pharmacological treatment acts on receptors and transmitter systems that extend beyond the circuits affected by the underlying pathophysiology (Figure 4). It can therefore improve function in one territory while altering activity and plasticity in another.

In Parkinson’s disease, dopamine replacement improves motor function in denervated territories, while its effects in relatively preserved circuitry can alter cognition, motivation and perception. Among the recognised adverse effects are impulse control disorders, which are particularly associated with dopamine agonist treatment [1]. Medication can improve one cognitive operation while impairing another, as shown by contrasting effects on task switching and reversal learning [104]. Psychosis also has disease-related determinants that interact with medication effects [2].

Levodopa supplies dopamine without reconstructing the axon terminal and feedback machinery that normally regulates its delivery. As buffering capacity declines, treatment-related fluctuations contribute to off periods and dyskinesia [105,106,107,108]. Continuous intestinal delivery reduces off time compared with oral treatment [109], illustrating the benefit of improving temporal delivery. Treatment can also be refined through receptor selectivity. The D1/D5 agonist tavapadon has demonstrated motor efficacy in a phase 3 trial [110]. This selectivity still spans compartments because D1-expressing populations occur in both the matrix and striosomes.

In schizophrenia, D2 blockade can reduce psychotic symptoms while interfering with dopamine-dependent function in other territories. Drug-induced parkinsonism illustrates this effect in motor circuitry. Treatment-related reductions in motivation or expression can resemble negative symptoms, while disorganisation may involve additional mechanisms [3,111]. Our framework predicts incomplete benefit when treatment corrects dopamine signalling but leaves the representations or connections on which it acts substantially impaired.

Clozapine’s efficacy in treatment-resistant schizophrenia, together with clinical occupancy studies, shows that antipsychotic benefit does not require sustained high striatal D2 occupancy [112,113]. Rapid D2 dissociation offers one possible explanation: less sustained occupancy could allow greater scope for dynamic dopamine modulation while retaining therapeutic benefit [3]. In preclinical models, sustained D2 blockade sufficient for antipsychotic-like effects has been proposed to drive dopamine neurons into depolarisation block, thereby interrupting their firing [114]. Within our framework, this proposed temporal mechanism complements clozapine’s broader receptor actions and regional effects. Molecular mapping adds evidence of regional differences in treatment response: chronic clozapine administration in mice produced transcriptional changes that varied by region and cell type, with a ventral bias and substantial matrix involvement [44]. These findings identify populations and territories in which treatment alters gene expression.

Pimavanserin provides a complementary clinical example. Its 5-HT_2A_ inverse agonist/antagonist action can reduce hallucinations and delusions in Parkinson’s disease psychosis without direct dopamine receptor blockade, and a pivotal trial found no significant worsening of motor function [115]. Differences in serotonergic responses between cortical projection classes in mice, together with apical 5-HT_2A_ localisation in primates, suggest ways that serotonin could influence the representations entering basal ganglia circuits [116,117]. These observations motivate tests of whether serotonergic treatment changes the representations that receive dopaminergic significance.

Muscarinic treatment provides a further example: xanomeline–trospium has demonstrated antipsychotic efficacy without direct D2 blockade [118]. Together, these treatments show that antipsychotic benefit can be achieved through different receptor mechanisms. Our framework asks which representations and pathways each treatment influences and how it changes dopamine dynamics. The therapeutic aim is to improve function in affected circuits while preserving selection and learning elsewhere. Anatomical distribution, receptor pharmacology and timing offer complementary routes towards this selectivity, whose circuit effects can be tested directly.

## 9. Limitations and Alternative Interpretations

An alternative to the proposed content–value weighting is that striosomes and the matrix represent similar combinations of information but differ primarily in timing, plasticity or access to downstream targets. Shared cortical inputs and overlapping action–outcome representations are compatible with this interpretation [30,71]. Recordings from striosome-enriched populations also show similar outcome-aligned responses on rewarded and unrewarded trials [15], arguing against a universal striosomal reward code. These interpretations can be distinguished experimentally. If compartment-resolved recordings reveal different temporal dynamics but no reproducible relative weighting of content and value, the computational interpretation would need revision, while the anatomical crossing would remain intact.

A second question concerns convergence. Striosomal inhibition, direct matrix input and SNr-mediated disinhibition can generate different dopamine responses depending on which synapses are recruited [30,39,42,52]. Anatomical proximity alone does not establish coordinated convergence on the same dopamine neuron. The unresolved question is how these pathways combine to regulate individual dopamine neurons. Failure to demonstrate the proposed convergence would challenge this feedback implementation without overturning the established compartment–receptor populations.

Axonal collateralisation further supports an organisation of overlapping, preferential routes [41]. D1/D2-coexpressing SPNs that project to GPe and modulate the motor effects of the principal populations add another source of interaction [45]. The four compartment–receptor combinations therefore describe the principal organisation rather than an exhaustive cellular taxonomy. Coexpressing neurons should be analysed as an additional population, with causal experiments testing how their recruitment modifies the activity and behavioural effects of the principal routes.

A third question concerns generalisation across species and regions. Compartmental organisation is well established beyond rodents, but the four routes, their relative strengths and their behavioural effects have been characterised most completely in mice [9,15,37,68,119]. Differences in nigral organisation and human striatal molecular specialisation indicate that local implementations may vary across species and territories [44,120]. In primates, for example, the ventral tegmental population that is anatomically distinct in rodents is displaced into the dorsal substantia nigra [120], so nigral subsystems do not map directly between species. Testing the proposed clinical mechanisms in humans therefore requires identifying the relevant populations and evaluating their molecular correspondence, projections and physiology separately.

The clinical applications concern proposed mechanisms within these circuits. Registration describes the appropriate association of content with value; its disruption is a computational hypothesis for schizophrenia, not an established cellular lesion. Disrupted apical integration is one candidate mechanism, while recurrent cortical, thalamic and striatal processes could produce similar errors. This formulation allows registration to be tested without assigning psychosis to a single neuronal class.

Our framework makes these alternatives experimentally distinguishable by separating receptor effects, route organisation, information weighting and regional pathology.

## 10. Testing the Framework: Predictions and Falsification

Table 2 connects the framework’s principal predictions with the experimental design each requires and the outcomes that would support or challenge each one. The proposed content–value distinction yields a central prediction: activity in matrix populations should support relatively better decoding of candidate identity, whereas activity in striosomal populations should support relatively better decoding of value. Tests should vary identity and value independently while controlling movement, arousal, reward history and sampling across compartments. The critical result is a compartment × information-type interaction consistent with this relative double dissociation. Its reproducible absence in adequately sampled, well-controlled tasks would directly challenge the content–value account.

Table 2 specifies measurable outcomes and results that support or challenge each prediction. A relative double dissociation requires both compartmental preferences, rather than an interaction driven by only one contrast. Minimum effect sizes should be justified from independent data, measurement reliability and the experimental question, with sample sizes planned for the required precision and multiplicity addressed where appropriate. The framework predicts directions and relationships but does not specify universal numerical effect sizes across species and paradigms.

Causal tests should ask whether changing the association between content and value changes which candidate acquires learned significance. For example, pairing the representation of one candidate with value-related activity associated with another should selectively transfer significance to the wrong candidate. Simultaneous measurements of behaviour, dopamine dynamics and compartment-resolved activity would distinguish this effect from general changes in movement or arousal. This tests registration as a computation without assuming that its disruption reproduces schizophrenia.

The feedback mechanism requires an anatomical and physiological test of convergence. Independently manipulating striosomal input and matrix input acting through SNr circuitry, while recording an identified dopamine neuron, would establish how the two routes interact. Synaptic mapping should verify the origins and direct or relayed connections of the inputs; recordings should establish their effects during and after activation. Failure to detect the predicted convergence under adequately sensitive conditions would challenge this implementation while leaving the four principal route biases intact.

Translation across species requires establishing the corresponding populations, connections and responses. Primate studies should test opposing striosomal D1 and D2 control of nigral dopamine and the pallidal relay of the D2 route; existing observations in monkeys and cats provide supporting elements [37,119,121]. Human tissue and sufficiently mature cortical–striatal–midbrain assembloids offer complementary anatomical approaches [122,123,124]. One example is testing whether striosomal terminals preferentially contact vulnerable ALDH1A1-positive ventral nigral neurons.

Human imaging can test whether the proposed distinctions appear at the level of regional activity and network organisation. Recent resting-state and task-based fMRI studies have identified different networks and relatively different cue- and movement-related responses in connectivity-defined striosome-like and matrix-like voxels [125,126]. These findings support further tests of information weighting without directly establishing the proposed content–value distinction. Connectivity-based parcellation [127], prospective high-resolution μ-opioid PET [10,128] and layer-resolved fMRI [129] provide complementary approaches. Their interpretation requires independent validation because current imaging does not resolve individual histological compartments or identify IT and PT neurons directly.

Network tests should ask whether content-related activity preferentially couples cortical–matrix-like–SNr regions, while value-related activity preferentially couples cortical–striosome-like–SNc regions. Cortical-depth profiles and separately localised nigral signals could help distinguish these relationships. Layer-specific functional connectivity and high-resolution imaging of nigral subregions provide methodological precedents [130,131]. Regional volume, partial volume effects and vascular contributions must be accounted for. A reproducible absence of the predicted information-dependent coupling, with validated proxies and adequate sensitivity, would challenge this human functional implementation. The sign of an fMRI correlation does not establish synaptic excitation or inhibition.

Relating these networks to dopamine release provides a further test of the controller account. Value-related activity in cortical–striosome-like networks should explain variation in regional striatal dopamine release beyond movement, arousal and activity in matrix-like networks. PET and fMRI can examine this relationship: previous studies have related amphetamine-evoked prefrontal dopamine release to separately acquired working memory activation and have linked striatal dopamine release to cortical activity during reward expectation using simultaneous PET–fMRI [132,133]. Complementary acquisitions in the same participants could test the predicted coupling. Its reproducible absence under adequately sensitive, anatomically validated conditions would challenge this extension. Correlations would not establish causal direction or resolve dopamine release within individual compartments.

Pharmacological trials can test how information weighting and network coupling change when dopamine availability or receptor signalling is altered. The key comparisons concern drug × compartment × information-type interactions, alongside corresponding changes in cortical–striatal–nigral coupling. Dopamine-enhancing and D2-blocking conditions provide examples; human fMRI has already shown modulation of reward prediction error signals by levodopa and haloperidol [134]. Interpretation requires measured target engagement and dose–response relationships, with drug-related vascular effects distinguished from neural modulation. Where PET is used, antipsychotic receptor occupancy must also be distinguished from displacement caused by endogenous dopamine.

Combination trials address whether treatments influence a shared downstream process or make separable contributions. When a shared process approaches saturation, combined effects may be subadditive; separable processes may retain additive effects when the outcome can capture both contributions. Dose–response comparisons, a prespecified model of additivity, and circuit measurements are needed to distinguish convergence from inadequate engagement or an outcome ceiling. For example, istradefylline enhanced the motor response to low-dose levodopa but added no benefit during an individually optimised levodopa infusion [135]. This illustrates the question without establishing compartment selectivity or formal subadditivity.

In schizophrenia, adjunctive trials could ask whether a treatment changes the same representations and dopamine feedback measures as the background antipsychotic or contributes through a different process. Opioid antagonism with naltrexone and treatment with purified cannabidiol provide examples beyond conventional D2 blockade. A small naltrexone trial found no symptomatic benefit, while cannabidiol improved positive symptoms in one adjunctive trial but not another [136,137,138]. Neither agent is striosome- or matrix-selective. Pairing clinical outcomes with the predicted information processing and coupling measures would therefore be necessary to relate their effects to this framework.

Antipsychotic combinations pose a similar question. They can increase antagonism at shared receptors or alter the balance of additional receptor actions, without separately targeting the two compartments. Adding risperidone to clozapine, for example, adds D2 and 5-HT_2A_ antagonism to clozapine’s broader receptor profile; one placebo-controlled trial found no additional symptomatic benefit [139]. Such a result alone cannot distinguish shared downstream action from failure to engage the process responsible for residual symptoms. Additivity or subadditivity becomes informative when interpreted alongside receptor engagement and circuit responses.

More selective tests require druggable targets whose expression differs between the relevant populations. Examples include μ-opioid receptors (OPRM1), enriched in striosomes but also expressed in other SPN populations [43,44]; CB1 receptors (CNR1), enriched in caudal dorsolateral striosomal circuits and striosome–dendron bouquets in mice [140]; and somatostatin SSTR2 receptors, localised to matrix SPNs and cholinergic interneurons in mice [141]. These are candidates for target development, not established compartment-selective treatments. Their protein distribution, accessible binding sites and functional effects require confirmation in the relevant human territory. Verified preferential engagement would permit a more direct test of the predicted separation between information weighting, behavioural selection and dopamine feedback.

Anatomical routing, information weighting and therapeutic convergence require distinct tests. Evaluating them separately allows each result to refine the framework precisely.

## 11. Conclusions

The striatum is organised by compartment as well as receptor identity. Reading these coordinates together connects the classical pathways for behavioural selection with striosomal pathways that regulate dopamine. The essential distinction is between acting on an output gate and acting on the signal that influences that gate. This anatomical distinction generates testable predictions about information weighting, feedback timing and treatment selectivity. The proposed actor–controller organisation (Box 2) brings established findings into one functional account, with distinct tests for each proposed functional consequence. A receptor label identifies part of the mechanism. Its position in the circuit explains what that mechanism can do.

Box 2 **Glossary.**

**Actor and controller:** The complementary roles proposed for the compartments. The matrix is the actor, releasing one candidate and suppressing its rivals through the output nuclei. The striosome is the controller: its output is not the selected action but the dopamine signal that biases, teaches and buffers the actor.

**Aberrant salience:** The attribution of significance to a stimulus or internal representation that does not warrant it; proposed as the phenomenological core of psychosis; here, it is placed at the binding of content to value rather than in dopamine magnitude alone.

**Cellular polarity:** The direction in which dopamine biases a spiny neuron’s intracellular signalling—D1 raising and D2 lowering cAMP–PKA. It does not specify invariant whole-cell excitation or inhibition; compartment changes how the same coupling is integrated.

**Content and value:** The two properties of a candidate. Content is its identity, timing and available response; value is its expected benefit, cost, urgency or contextual significance. We propose that matrix populations weight content and striosomal populations weight value—a relative bias within overlapping inputs, not a strict segregation of afferent lines.

**Dendron bouquet:** The specialisation in which striosomal axons entwine bundled ventral dendrites of SNc dopamine neurons descending into the pars reticulata, providing the anatomical substrate for distal, GABA_B_-recruiting inhibition.

**IT neurons:** Intratelencephalic corticostriatal projection neurons that project within the telencephalon. Their proposed relative weighting toward the matrix is provisional and may vary by region, species and task; IT inputs reach both compartments.

**Orthogonality:** Architecturally separate coordinates that cross-classify the same neuronal population; not a claim of equal proportions, absolute statistical independence, or exclusively single-receptor expression. Afferent route and cell class feed compartment as correlated biases, not as a third axis.

**PT neurons:** Pyramidal tract corticostriatal projection neurons of deep layer 5 that project subcerebrally and send corticostriatal collaterals. Their apical dendrites can integrate distal inputs. Their proposed relative weighting toward striosomes is provisional; PT inputs reach both compartments.

**Registration:** The appropriate binding of a candidate’s content to its value before selection. A computational relation with several possible cellular implementations, of which apical–somatic integration in layer-5 PT neurons is one candidate; its failure is proposed as the circuit-level form of aberrant salience.

**Route sign:** The net effect a route delivers to its final target, set by the number of inhibitory links between the striatal neuron and that target. It inverts between compartments because the targets differ, not because the receptor does.

**Striosomes:** Chemically distinct striatal compartments, enriched in μ-opioid receptor and poor in acetylcholinesterase, occupying roughly 10–15% of the striatal volume in a three-dimensional labyrinth through the surrounding matrix [142], and an important source of inhibitory input to nigral dopamine neurons; matrix and exo-patch populations also contribute [30,42].

## Figures and Tables

**Figure 1:**
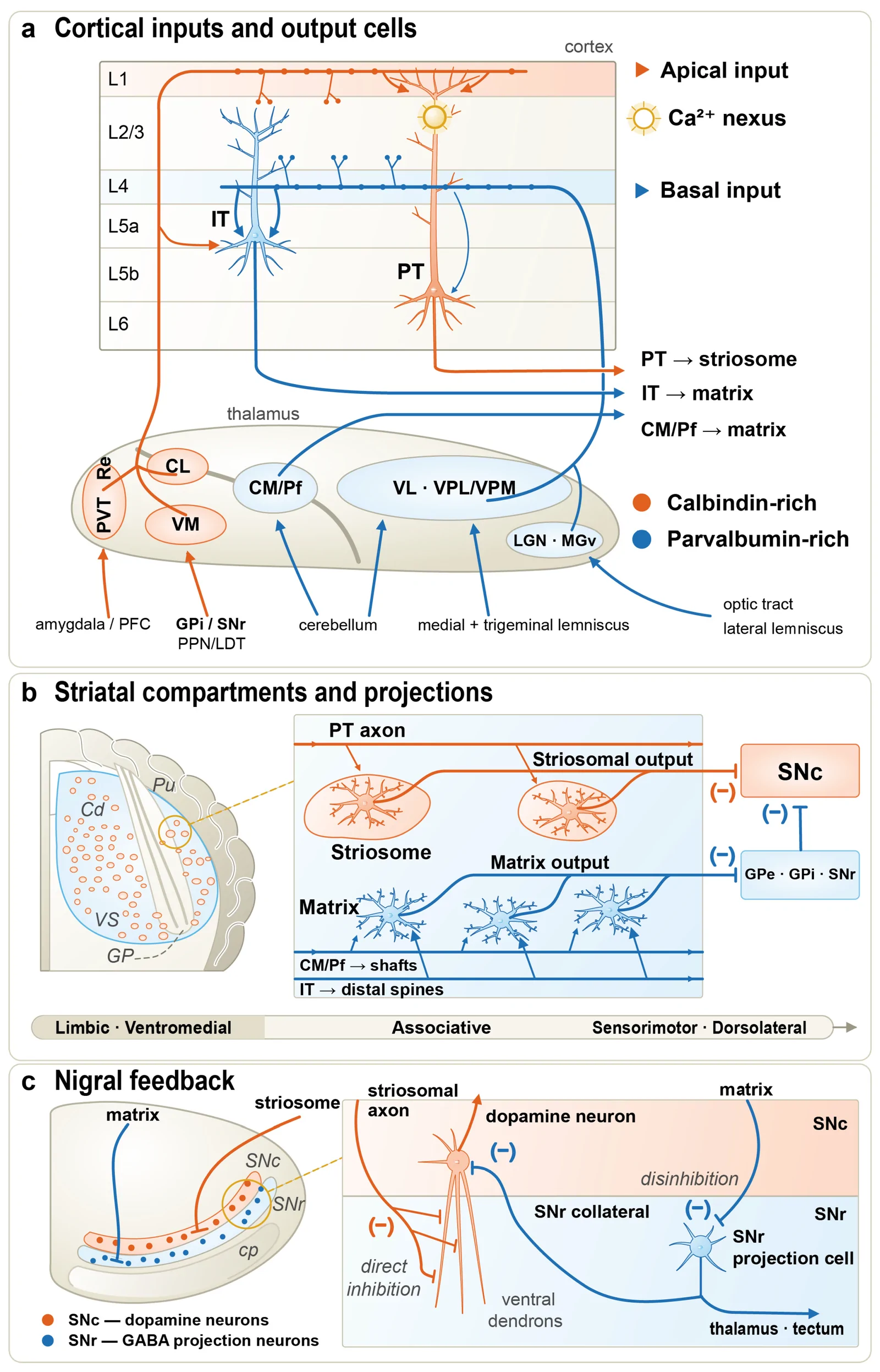
Cortical inputs, striatal compartments and nigral feedback. Orange and blue distinguish the pathways and populations labelled in each panel: apical and basal input in (**a**), striosomal and matrix circuitry in (**b**), and dopamine and SNr projection neurons in (**c**). Colours identify the illustrated populations and pathways; they do not imply exclusive content or value coding. Arrowheads show projection direction; bar-ended contacts indicate inhibition. Gold marks the calcium nexus and the regions enlarged in the insets. L1–L6, cortical layers 1–6; L5a/L5b, sublaminae of layer 5; IT, intratelencephalic; PT, pyramidal tract; Ca^2+^, calcium ion; PVT, paraventricular thalamic nucleus; Re, nucleus reuniens; CL, central lateral nucleus; VM, ventromedial nucleus; CM/Pf, centromedian–parafascicular complex; VL, ventral lateral nucleus; VPL/VPM, ventral posterolateral/posteromedial nuclei; LGN, lateral geniculate nucleus; MGv, ventral division of the medial geniculate nucleus; PFC, prefrontal cortex; PPN/LDT, pedunculopontine/laterodorsal tegmental nuclei; Cd, caudate; Pu, putamen; VS, ventral striatum; GP, globus pallidus; GPe/GPi, external/internal globus pallidus; SNc/SNr, substantia nigra pars compacta/reticulata; GABA, γ-aminobutyric acid; cp, cerebral peduncle.

**Figure 2:**
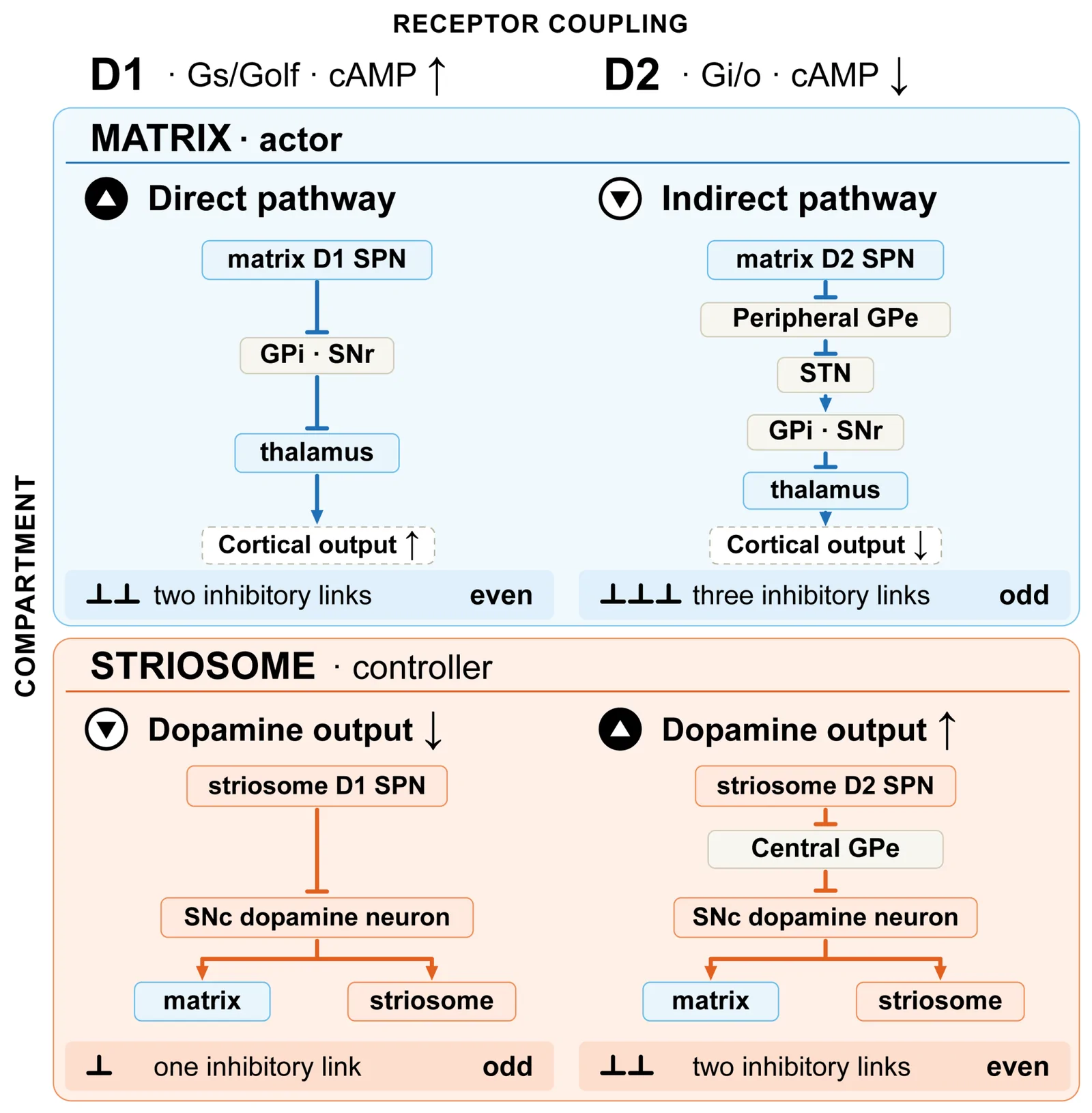
The receptor sets the switch—the route sets the sign. Receptor and compartment identity define four principal populations of striatal spiny projection neurons (SPNs): matrix D1, matrix D2, striosomal D1 and striosomal D2. The diagrams show the effects of increased SPN activity, distinct from dopamine receptor activation; blue denotes matrix routes; orange denotes striosomal routes. Bars indicate inhibition; arrowheads indicate excitatory or dopaminergic projections. Circled upward and downward triangles indicate net increases and decreases in the specified output. Inhibitory link counts summarise the sign of each simplified pathway. D1/D2, dopamine receptor types 1 and 2; Gs, stimulatory G protein; Golf, olfactory-type stimulatory G protein; Gi/o, inhibitory G-protein family; cAMP, cyclic adenosine monophosphate; SPN, spiny projection neuron; GPe/GPi, external/internal globus pallidus; STN, subthalamic nucleus; SNc/SNr, substantia nigra pars compacta/reticulata.

**Figure 3:**
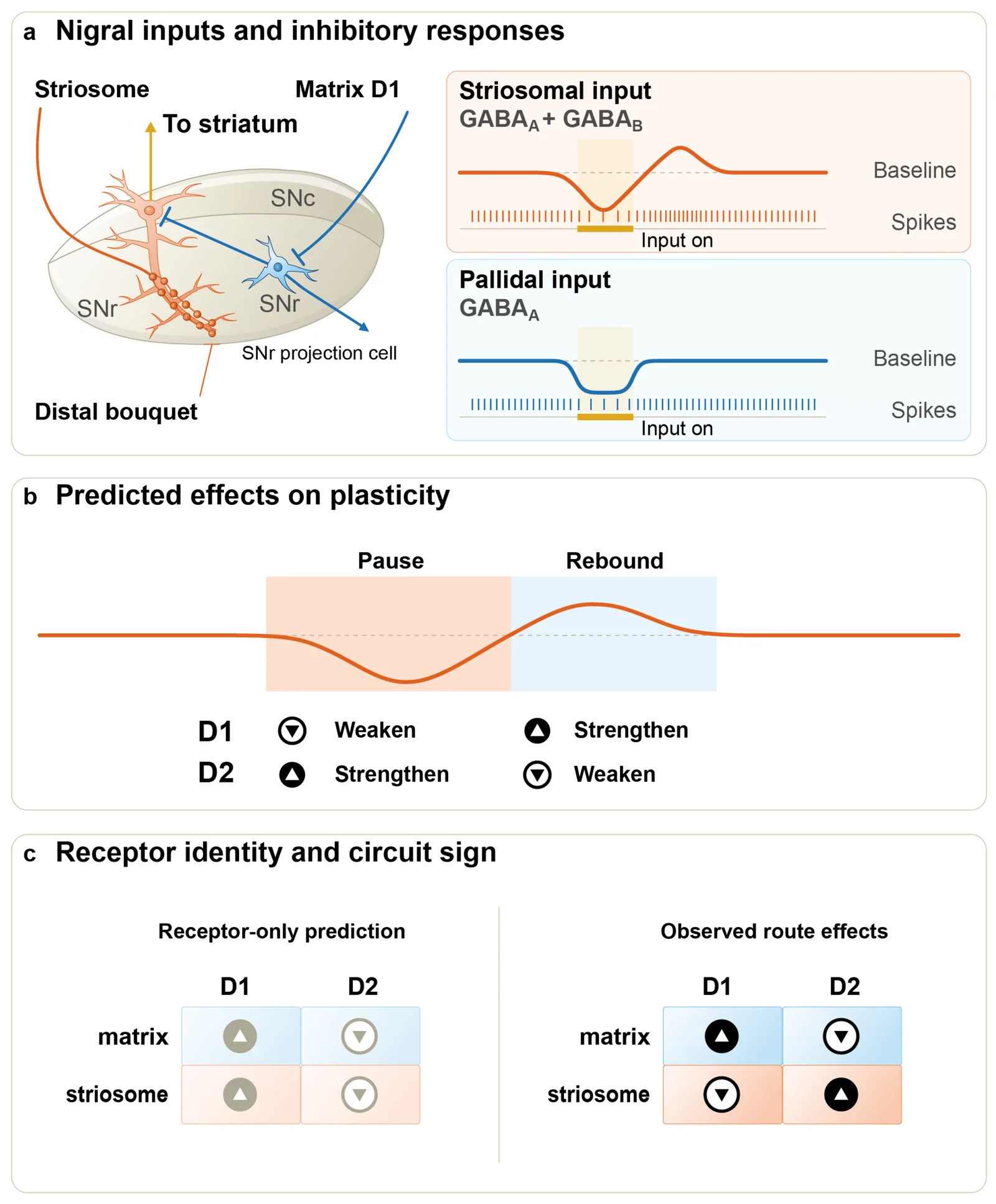
Nigral feedback, dopamine dynamics and predicted plasticity. (**a**) Striosomal inhibition of dopamine neuron dendrites (orange) and matrix D1-mediated disinhibition through SNr collaterals (blue) provide complementary feedback routes [36,38,39]. Their coordinated convergence onto the same dopamine neuron is proposed; gold marks dopamine output. Right: Striosomal input recruits GABA_A_ and GABA_B_ components and can produce rebound firing, whereas pallidal input predominantly recruits GABA_A_ receptors without comparable rebound under the tested conditions [42]. The pallidal response shown on the right was not measured from the SNr connection illustrated on the left. Traces are drawn to measured values rather than recordings: striosomal inhibition reduces firing to 37.6% of baseline, and the first interspike interval after release reaches 5.3 Hz, against 2.8 Hz for pallidal input [42]; gold bars and shading mark stimulation, and dashed lines indicate baseline firing. (**b**) The pause–rebound firing sequence is predicted to weaken then strengthen synapses in D1-expressing striatal SPNs, with the reverse in D2-expressing SPNs [48]. These plasticity predictions require testing in each compartment. Shading denotes temporal phases, not compartments. (**c**) A receptor-only scheme assigns the same sign across compartments. Observed route effects combine classical matrix effects on behavioural output with opposing striosomal D1 and D2 effects on dopamine output in mice [15]. Circled triangles indicate strengthening or weakening in (**b**) and positive or negative output effects in (**c**). D1/D2, dopamine receptor types 1 and 2; GABA_A_/GABA_B_, ionotropic/metabotropic γ-aminobutyric acid receptors; SNc/SNr, substantia nigra pars compacta/reticulata.

**Figure 4:**
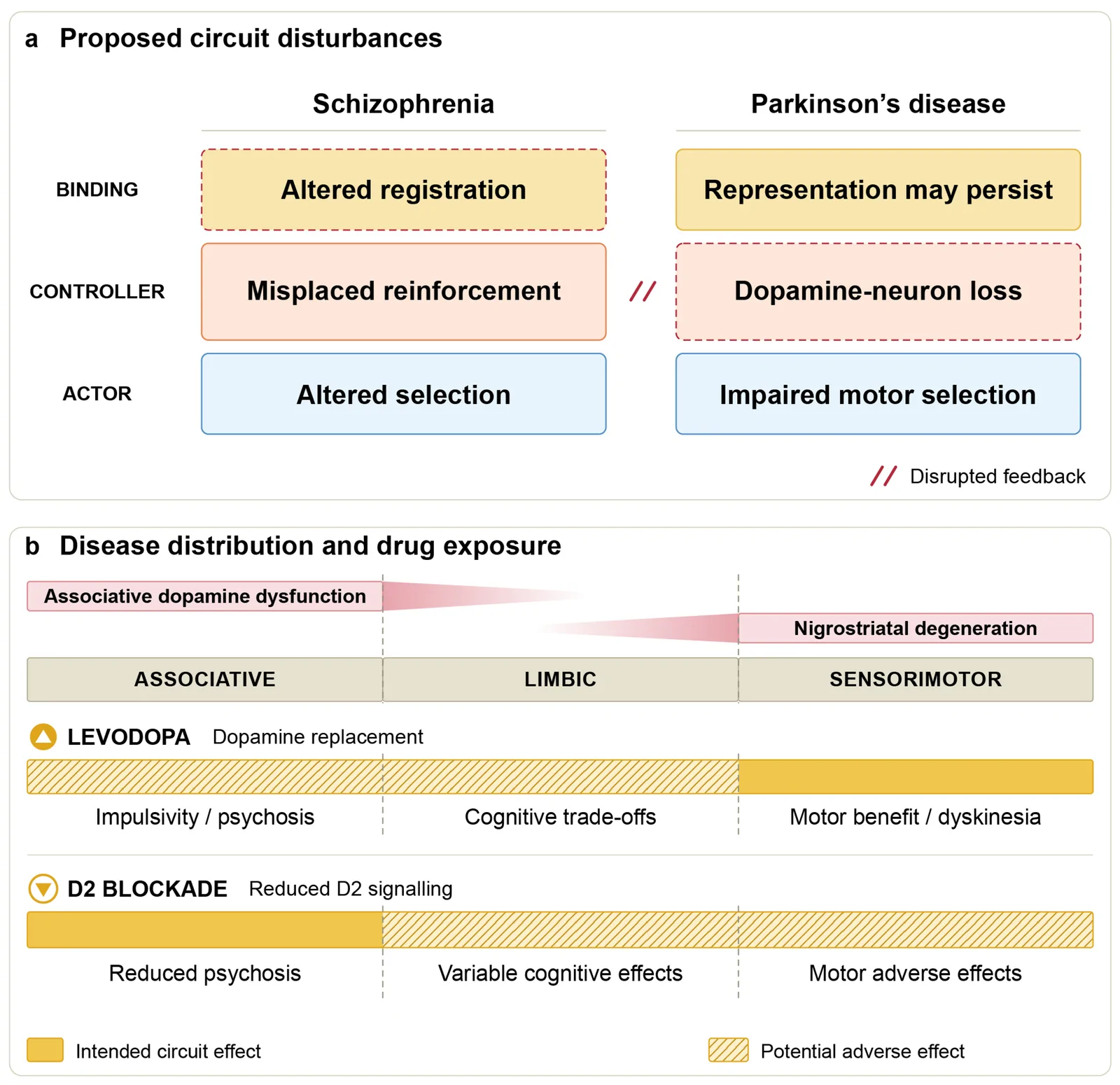
Circuit disturbances, disease distribution and drug exposure. (**a**) Binding (registration), controller and actor describe the proposed circuit relationships, coloured gold, orange and blue. In schizophrenia, the dashed binding box represents a hypothesis in which a candidate acquires inappropriate significance. Misplaced reinforcement and altered selection illustrate possible consequences without establishing a cellular origin or necessary causal sequence. In Parkinson’s disease, nigrostriatal neuron loss impairs dopamine-dependent selection and feedback capacity, indicated by the dashed controller box and double slash. “Representation may persist” isolates this comparison without implying that representations are uniformly spared. The rows are circuit relationships rather than clinical stages. (**b**) Pink bands contrast associative striatal dopamine dysfunction in schizophrenia [96,97] with the sensorimotor emphasis of nigrostriatal degeneration in Parkinson’s disease. The columns are conceptual territories; their order, widths and tapers do not specify anatomical boundaries or disease progression. Gold treatment bars show the broader reach of dopamine replacement and D2 blockade. Circled upward and downward triangles distinguish increased dopamine availability from reduced D2 signalling. Solid fill marks the intended therapeutic territory; hatching marks potential adverse effects elsewhere. Benefit and adverse effects can coexist within the intended territory, as illustrated by motor improvement and dyskinesia. Dopaminergic treatment can improve movement while impairing particular cognitive operations in relatively preserved circuitry [104]. Dopaminergic psychiatric adverse effects and D2-blockade-associated motor effects provide the clinical comparison [1,2,3]. Drug effects vary with receptor distribution, dose, timing and disease state; the bars do not imply equal effects across territories or exclusive localisation of the listed outcomes. Psychosis in Parkinson’s disease also has disease-related determinants. Both disorders involve pathology beyond this dopaminergic comparison. D2, dopamine receptor type 2.

**Table 1:** Five perspectives on basal ganglia function and their integration within the compartment–receptor framework.

The Scheme	What It Saw Truly	What It Took for the Whole	Where It Falls
**Dopamine, depleted and blocked** (1960–1976) [16,17,18,19,20,21,22]	That one transmitter, lost from one nucleus, produces one syndrome, and returning it returns movement, and that potency against psychosis tracks affinity at its receptor	A quantity, read from both ends at once; too little in one clinic, too much in the other	The **receptor axis**—one axis, two poles, and neither clinic recognised it as the same axis
**Parallel, largely segregated loops** (1986) [23]	That the structure performs one operation and repeats it on movements, goals and affect alike	Territory as the organising partition, so that what differed between loops was taken for what mattered	Not a coordinate at all; territory **scales** the crossing; it sets which candidate is at stake, not which operation runs
**Direct and indirect pathways** (1983–1990) [4,5,6,7]	That polarity is generated inside the striatum, by receptor identity, and that two populations oppose one another	Two pathways; the count was made in the matrix and read as the whole striatum	The **receptor axis**, correctly—but counted in one compartment only
**Striosome and matrix** (1978–1989) [9,12,13]	A second partition of the same neurons, chemically and connectionally independent of the first	A compartment: a place, a mosaic, a curiosity of histochemistry	The **compartment axis**—found, but named as a region rather than as a coordinate
**What the dopamine signal carries** (1991–2009) [24,25,26,27]	That the signal is structured in time and in meaning: tonic against phasic, prediction error, salience	One code on one line, as though the signal had a single source	The **output** of the striosomal controller, described without its controller

Five schemes, six decades, each shaped by a different question and method. Each reveals a different aspect of basal ganglia function. Reading compartment and receptor identity together brings them into one framework: dopamine deficiency and receptor blockade describe changes in signalling; direct and indirect pathways describe routes; striosomes and the matrix distinguish compartments; parallel loops organise these relationships across territories; and dopamine coding describes the signals they help generate. Their integration is the argument of this Perspective.

**Table 2:** Predictions and tests of the two-coordinate framework.

Prediction	Experimental Approach	Supporting Result	Result Challenging the Prediction
**Content–value double dissociation.** Matrix activity preferentially represents candidate identity; striosomal activity preferentially represents value.	Simultaneous recording of identified matrix and striosomal neurons during a choice task in which candidate identity and value vary independently (e.g., calcium imaging).	Matrix activity more strongly represents candidate identity, whereas striosomal activity more strongly represents value.	No meaningful compartment preference, or a reproducible reversal of the predicted pattern.
**Registration is causal.** Reassigning value from one candidate to another transfers learned significance to the wrong candidate.	Candidate–value learning followed by experimentally induced mispairing of candidate and striosomal value signals (e.g., through precisely timed optogenetic stimulation). Subsequent choice behaviour tests whether the mismatched value is transferred to the paired candidate.	The candidate paired with the mismatched striosomal value signal acquires the predicted change in choice preference, despite unchanged actual reward contingencies.	The mismatched striosomal value signal does not selectively alter preference for the paired candidate or produces only a general change in choice.
**Feedback convergence.** Striosomal D1 input and SNr-relayed matrix D1 input exert opposing effects on the same dopamine neuron.	Independent manipulation of striosomal D1 and SNr-relayed matrix D1 inputs while recording from the same identified dopamine neuron, with anatomical verification of each pathway (e.g., whole-cell patch-clamp in acute midbrain slices, pathway-specific optogenetic stimulation and post hoc tracing or histological confirmation).	Striosomal D1 input suppresses firing while SNr-relayed matrix D1 input increases firing in the same dopamine neuron, with anatomical and physiological evidence confirming the two pathways.	The two pathways do not produce opposing effects in the same dopamine neuron, or the matrix effect persists when the SNr relay is disrupted.
**Cross-species translation.** Primate striosomal D1 and D2 pathways oppose one another in controlling nigral dopamine.	Selective manipulation of striosomal D1 and D2 pathways in non-human primates, with measurement of nigrostriatal dopamine (e.g., pathway-specific viral targeting with chemogenetic or optogenetic manipulation and a dopamine sensor).	Activating striosomal D1 neurons decreases dopamine-sensor signals, whereas activating striosomal D2 neurons increases them in the same nigrostriatal territory.	Striosomal D1 and D2 manipulation does not produce opposite dopamine changes, or the effects are inconsistent or not confined to the anatomically verified nigrostriatal pathway.
**Human information-weighted coupling.** Content-related cortical activity preferentially couples with matrix-like striatal and SNr regions, whereas value-related cortical activity preferentially couples with striosome-like striatal and SNc regions.	High-resolution task-based fMRI with independently validated striosome- and matrix-like compartment proxies and network localisation, during a task in which candidate identity and value vary independently. Prespecified connectivity analyses test whether content- and value-related cortical activity preferentially couples with the predicted striatal and nigral regions.	Content-related cortical activity couples more strongly with matrix-like striatal and SNr regions, whereas value-related cortical activity couples more strongly with striosome-like striatal and SNc regions.	Content- and value-related cortical activity show no preferential coupling with the predicted matrix-like–SNr and striosome-like–SNc networks, or they show the opposite pattern.
**Human dopamine-release coupling.** Value-related activity in striosome-like regions predicts regional dopamine release more strongly than activity in matrix-like regions.	Simultaneous PET–fMRI during value-based task blocks, relating value-related activity in striosome-like and matrix-like regions to regional dopamine release measured with dopamine-sensitive PET, while controlling for movement and arousal.	Regional dopamine PET signal is more strongly associated with value-related activity in striosome-like than matrix-like regions across task blocks.	Dopamine signal does not preferentially track value-related activity in striosome-like regions or instead tracks matrix-like activity.
**Pharmacological convergence.** Drug combinations should interact according to where they act in the two-coordinate architecture: treatments converging on the same pathway should show redundancy, whereas treatments acting on complementary content- and value-related pathways may show synergy.	Factorial or within-subject pharmacological study during clinically appropriate treatment conditions, using a reinforcement learning task that independently varies action and value (e.g., a reward-modulated Go/No-Go task). Compare treatments predicted to converge on the same pathway with those affecting complementary circuit coordinates against a prespecified additivity model.	Treatments converging on the same pathway show less-than-additive effects, whereas treatments acting on complementary content- and value-related pathways show additive or synergistic effects.	Treatment combinations show similar effects regardless of whether they converge on the same pathway or act on complementary content- and value-related pathways.

## Data Availability

No new data were created or analysed in this study. Data sharing is not applicable to this article.
